# The Effect of Antiplatelet Drugs on the Patency Rate of Arterio-venous Fistulae in Hemodialysis Patients

**Published:** 2010

**Authors:** Mohsen Rouzrokh, Mohammad Reza Abbasi, Ali Reza Mirshemirani, Mohammad Reza Sobhiyeh

**Affiliations:** a *Department of Pediatric Surgery and Pediatric Surgery Research Center,Mofid Children’s Hospital, ShaheedBeheshti University of Medical Sciences,Tehran, Iran.*; b * Department of Adult Nephrology, Imam Khomeini Hospital, Tehran University of Medical Sciences, Tehran, Iran*.; c * PediatricSurgery Research Center, Mofid Children’s Hospital, ShaheedBeheshti University of Medical Sciences, Tehran, Iran.*; d * ShaheedBeheshti University of Medical Sciences, Tehran, Iran.*

**Keywords:** Patency rate, Arterial-venous fistulae, Aspirin, Dipyridamol

## Abstract

Although arterialovenous fistulae (AVF) is considered to be vital for chronic kidney disease (CKD) patients, but they may cause complications and problems. For instance they may fail soon after their creation. The most important cause of failure in these cases is intrafistula thrombus formation.

Whereas anti-platelet drugs are not routinely used after fistulae creation, we conducted this study to determine the effect of these drugs (aspirin and dipyridamol) on the patency of AVFs. From Sep 2003 to Aug 2007, all CKD patients who needed AVF for hemodilysis were included in our study.

After fistulae creation, they were randomly divided in 3 groups. The first group was received aspirin and the second one with dipyridamol and the third one was the control group that received placebo. Each group consisted of 130 patients. Exclusion criteria were bleeding tendency, active peptic ulcer disease, pregnancy, lactation, use of anticoagulant and or non steroidal anti-inflammatory drugs, hepatic insufficiency and history of significant side effects from aspirin or dipyridamol. The patency of AVF in the control, aspirin and dipyridamol groups were obtained 69.2%, 70.8% and 75.4% respectively. Although the patency in the aspirin and the dipyridamol group were 1.6% and 6.2% more than the control group, but there was no statistically significant difference between them and placebo (The p-value was 0.892 for the aspirin group and 0.332 for the dipyridamol group). Our study showed that neither the aspirin nor the dipyridamol can be effective on the patency of AVF after 72 h even within six months period.

## Introduction

Safe, reliable and durable vascular access is essential for successful hemodialysis. Long term patency rates demonstrate that native AV fistulae has the best outcome compared to other methods *e.g. *synthetic grafts and double lumen sialistic catheter. Autogenous AVF also has lowest cost and lowest infection rate (1-3). The patency rate of AVFs in most parts of the world is 60-70% in the first year and 50-60% in the second year. The Primary failure rate of an AVF ranges between 20–54% during the first six months (4, 5). Ineffective AVF and complications of AVF is one of the most problems of chronic kidney disease (CKD) patients. Some of the complications are thrombosis, infection, stenosis, aneurysm formation and distal limb ischemia (6). A team approach with nephrologists, surgeons, interventionalists, the patient, and the nursing staff, as well as the primary care staff is essential to preserve the vasculature for eventual fistula placement (7, 8). This is to start a policy to preserve access sites and to allow adequate time for planning, creation and maturation of the vascular access. The planning stage involves examination and pre-operative vascular mapping. The upper extremity AVF should be the preferred access and should be placed as distal as possible. The wrist radial-cephalic (RC) AVF is the first option for access creation. Any staff involved in handling vascular access or cannulating veins in renal patients should be adequately trained and be in a continuous training scheme for access management. The patient with chronic kidney disease should have a declared plan for preserving the vascular access and potential access sites (8).

Besides thrombosis and failure of maturation as the most factors for the primary failure rate of an AVF, patients in groups of elderly, female sex, underlying vascular disease and diabetes have higher failure rate (2-4, 9-11). It is recognized that 85% of AVF failures may be due to thrombosis. The most important predisposing factors for thrombosis are the size of the vein and artery, the skill of the surgeon creating the access. Other mechanisms of thrombosis that are less common include arterial stenosis, hypertension, increased hematocrite, and hypovolemia and hypercoagulative status (12).

Platelet activation from endothelial injury may play an important role in stimulating platelet aggregators such as thromboxane A2, in addition to directly stimulating vascular intimal proliferation. Therefore the therapeutic potential of antiplatelet agents including aspirin, sulfinpyrazone, dipyridamole, and ticlopidine might be useful (13-16). Antiplatelet agents often used the treatment of coronary artery diseases, peripheral vascular diseases, and cerebrovascular diseases (17). These drug use indicators are intended to measure specific aspects of health providers and drug use in a hospital or health center (18). However, safety concerns regarding the use of these agents have prompted the need for safe and effective base on treatment goals (19). The aim of this study is to evaluate the effects of two anti platelet drugs (aspirin and dipyridamol) on AVF patency rate. 

## Experimental

*Methods*

501 ESRD patients who needed hemodialysis were enrolled in the study. 111 patients were excluded by the end of the study due to fail of AVFs function within the first 72 h after the surgery, fail of follow up or drugs disadventages. These failures are mainly the result of technical problems during the operation.

Time for the first cannulation of the AVF was 2 months after AVF creation. These patients underwent fistulae creation surgery in Taleghani Hospital, Kermanshah, Iran from December 2003 to August 2007 by the same group of surgeon. Examination and pre-operative vascular mapping was done by application of a proximal tourniquet. Vein diameters of > 2–2.5 mm have been considered for this study with cephalic vein at the wrist as the first choice and the cubital veins as the second. The cephalic vein diameter increase after application of a proximal tourniquet is an important predictor of success. The AVFs fashioned in the snuff box (231 of all) were End to side anastomosis of cephalic vein to radial artery and AVFs of arm (remaining 159) were side to side anastomosis of cephalic or basilica vein to brachial artery. Patients were randomly divided into 3 groups (130 in each). The first group (78 females and 52 males) were treated with a daily 100 mg of Aspirin. The second group (65 women and 65 men) were treated with a daily 75 mg of dipyridamol. The third group (65 women and 65 men) were treated with placebo. All patients received anti-platelet drugs for at least 6 months. Aspirin and dipyridamol was made by one of Iranian companies. 

The patients with known bleeding disorder, pregnancy, lactation, active or suspected bleeding tendency, active peptic ulcer disease,severe hepatic insufficiency, already receiving anti-coagulation and regular non-steroidal anti-inflammatory (NSAIDS) agents drugs and significant side effects of drugs were excluded. Patency in the first 72 h post surgery was determined by the presence of a bruit over the site of the anastomosis, with standard stethoscope. The patency of the AVF at two months after randomization evaluated by the presence of an audible bruit and palpation the characteristic thrill and the intravascular pressure over the site of the AVF. Time to first cannulation of the AVF (time taken as surgery until the first attempt at access cannulation) was 2 month. Failing AVF was considered with decreased thrill or pulse alone, elevated venous dialysis pressures, no bruit over AVF site, decreasing dialysis dose delivery, decreasing flow rates, and difficulty in cannulating. At least all of the patients were followed for functional patency 6 months to 2 years and this was defined as the ability to use the AVF for dialysis to the satisfaction of the clinician(s). No surgical or radiological intervention was done for correction of AVF.

We collected all data on age, gender, and underlying disease, site of the AVF, extremity dominancy, and effects of aspirin and dipyridamol on patency rate of the AVF.

The data was processed by the SPSS 16 software and statistical analysis was performed by Chi-square test and fisher’s exact test.

## Results and Discussion

At least in six month period, 390 patients out of 501 (130 cases randomized in each group) remained and 111 patients were excluded, because they had failed to follow up, whose AVFs had failed within the first 72 h after the surgery or drugs discontinuity. 

In 390 patients (179 males, 211 females), 22 patients were aged less than 25 years old, and 74 cases were above 66 years of age and the rest were aged between these ages.

Among different age groups the best patency rate belonged to patients between 46-65 years, and the worst patency rate was for patients above 66 years old, possibility related to the vascular atherosclerosis that is common in elderly patients. Overall there was no statistically significant difference between differ age groups (Table 1).

**Table 1:** Fistula (AVF) patency rate according to different age groups

**AVF patency in age group**	**aspirin (%)**	**p** ***-*** **value**	**dipyridamol (%)**	**p-value**	**control (%)**
< 25	10/10 (100)	0.2308	8/9 (88.9)	0.4545	2/3 (66.7)
26 - 35	11/11 (100)	0.4762	13/13 (100)	0.1993	9/11 (81.8)
36 - 45	15/27 (55.5)	0.2870	21/24 (87.5)	0.4361	15/20 (75)
46 - 55	25/30 (83.3)	0.6669	23/28 (82.1)	0.7694	25/33 (75.8)
56 - 65	24/30 (80)	0.9698	24/33 (72.7)	0.7624	26/34 (76.5)
66 >	7/22 (31.8)	0.6447	9/23 (39.1)	0.8357	13/29 (44.8)

There was no statistically significant difference between the patency rates among different genders and different anatomic places of anastomosis (left wrist, left arm, right wrist and right arm) either (Table 2).

**Table 2:** AVF patency rate according to gender in aspirin, dipyridamol and control groups

**AVF patency in Gender **	**aspirin (%) **	**p** ***-*** **value **	**dipyridamol (%) **	**p-value **	**control (%) **
Female	58/78 (74.4%)	0.2028	50/65 (76.9%)	0.2671	41/65 (63%)
Male	40/52 (76.9%)	0.8463	48/65 (73.8%)	0.9515	49/65 (75.4%)

The patency rate of the AVF in two groups of aspirin and placebo is higher in male than female probably due to better vasculature (Table 2).

The most important underline disease was diabetic mellitus and hypertension (diabetic mellitus in 96 cases, Hypertension in 114 cases and both hypertension and diabetic mellitus in 110). Others had not important underlying diseases.

The lowest patency rate was related to the combination of hypertension and diabetes (Table 3). 

**Table 3:** AVF patency rate according to underlying disease in aspirin, dipyridamol and control groups

**AVF patency in each underlying disease **	**aspirin (%) **	**p** ***-*** **value **	**dipyridamol (%) **	**p-value **	**control (%) **
DM	14/20 (70%)	0.9531	28/35 (80%)	0.5065	29/41 (70.7%)
HTN	21/33 (63.6%)	0.5911	30/38 (78.9%)	0.6486	31/43 (72.1%)
HTN+DM	29/45 (64.4%)	0.7958	22/36 (61.1%)	0.8386	17/29 (58.6%)

The high AVF patency rate related to left wrist and elbow in the two groups mentioned is lower than the patency rate related to the right elbow (Table 4).

**Table 4:** AVF patency rate according to anatomic location in aspirin, dipyridamol and control groups.

**AVF patency in anatomic location **	**aspirin (%) **	**p** ***-*** **value **	**dipyridamol (%) **	**p-value **	**control (%) **
Left wrist	38/52 (76%)	0.4808	42/56 (75%)	0.3420	33/51 (64.7%)
Left elbow	32/45 (71.1%)	0.8743	31/40 (77.5%)	0.7928	30/40 (75%)
Right wrist	16/23 (69.6%)	0.9798	17/23 (73.9%)	0.9639	18/26 (69.2%)
Right elbow	6/10 (60%)	0.6850	8/11 (72.8%)	1	9/13 (69.2%)

The AVF were created in dominant upper extremity in 121 cases (15 cases in right and 106 cases in left) and in non-dominant extremity for 284 cases. There was no significant difference in the patency rate of AVF between dominant and non-dominant extremity (Table 5).

**Table 5:** AVF patency rate according to limb dominancy in aspirin, dipyridamol and control groups.

**AVF patency in dominant extremities **	**aspirin (%) **	**p** ***-*** **value **	**dipyridamol (%) **	**p-value **	**control (%) **
Dominant	30/42 (71.4%)	0.8756	29/40 (72.5%)	0.9365	26/39 (66.6%)
Non-dominant	60/88 (68.1%)	0.9331	63/90 (76.6%)	0.3042	63/91 (69.2%)

The patency rate of AVF in the control, aspirin and dipyridamol groups was obtained 69.2%, 70.8% and 75.4%, respectively. Although the patency rate in the aspirin group and the dipyridamol group was 1.6% and 6.2% more than the control group respectively but there was no statistically significant difference between them and the placebo (with a p-value of 0.892 for the aspirin group and 0.332 for the dipyridamol group) (Table 6). The most side effects of the aspirin was gastrointestinal bleeding, nausea and stomach pain and about the dipyridamole was nausea and vomiting in up to 6% of patients.

**Table 6:** Distribution of fistulae status in the aspirin and dipyridamol group six month after operation

**Gro** **up**	**aspirin (%) **	**p** ***-*** **value **	**dipyridamol (%) **	**p-value **	**control (%) **
**Fistulae status**					
Patency	92/130 (70.8%)	0.892	98/130 (75.4)	0.332	90/130 (69.2)

Early referral of CKD patients to the nephrologists and/ or vascular surgeon is strongly recommended. This is to start a policy to preserve access sites and to allow adequate time for planning, creation and maturation of the vascular access. The vein diameters of <1.6 mm have been associated with AVF failure (19), while good patency rates were obtained in patients with radio-cephalic AVFs where the diameter of the cephalic vein at the wrist was >2–2.6 mm and 2 mm or larger for arteries (20). An autogenous fistula requires at least 6 weeks for maturation before it can be used. For these reasons, it is recommended that the fistula is created at least 2–3 months before the earliest likely date for starting haemodialysis (19, 20). The preoperative criteria for creating a successful upper extremity AVF were diameters of 2 mm or larger for arteries and 2.5 mm for veins (21-22).

Venous preservation with additional handgrip exercise may enhance the quality and diameters of arteries and veins for fistula creation (23, 24). Protection of forearm and central vein vasculature from repeated cannulation injury, preoperative venous mapping to optimize the location and type of fistula placement and to rule out possible outflow obstruction, adequate maturation time, and dialysis staff expertise with fistula cannulation (25). All of these advices were attended in our series. According to our study, except the age of the patient that is important in patency rate of the AVF (which in young patients are better than it is in older patients), gender and site of the AVF were not significant (Tables 1, 2, 4). It seems the existence diabetes and hypertension could be worsening AVF survival (Table 3). Dominant upper extremity has more muscles activity and it may cause a better quality vascular of AVF creation than non dominant one, although no significant difference has been found in this study (Table 5). 

One of the most causes for AVF failure is thrombosis. Platelet activation from endothelial injury may play an important role in stimulating platelet aggregators, and thrombus forming. 

Aspirin has been chosen as it has well-established anti-platelet effects, aspirin’s anti-platelet effect is mediated by the inhibition the platelet enzyme cyclo-oxygenase resulting in blockade of the synthesis of the pro-aggregatory vasoconstrictor and thromboxane A2. Low dose of aspirin doubles the risk of serious gastrointestinal bleeding (26) and the theoretical risk may be higher in patients with CKD because of the presence of uremic induced impairment of haemostasis. In some studies a significant elevation in bleeding times in patients on haemodialysis treatments administered a single dose of aspirin was shown, but surprisingly little evidence-based clinical trial data is available in this population (27-29). Dipyridamol is a vasodilator that inhibits platelet function by inhibiting adenosine uptake and cyclic GMP phosphodiesterase activity (28).

No trial with a follow up longer than 36 months demonstrated a beneficial effect of anti platelet treatment to increase patency rate of AVF. In this study, drugs were prescribed at the most period of 2 years. In another study, it was concluded that consistent use of aspirin may be beneficial for AVF survivals among incident hemodialysis patients (15). On the other hand a cohort study demonstrated that treatment with antiplatelet medications was associated with significantly worse AVF patency rate (30, 31). 

In this study, the rate of AVF failure after six months of operation was 30.8%, 29.2% and 24.6% in control, aspirin and dipyridamol groups, respectively. No significant difference was observed as it is shown. However, long term effect of antiplatelet agents on AVF patency needs further investigation.

It was concluded that the patency rate does not depend on gender, however, but the patient’s personal factors such as age and underlying diseases are important. The young patients without underlying diease (diabetes and hypertension) have longer patency period. There was no significant difference in different places of the AVF. In conclusion, neither the aspirin nor the dipyridamol had any effect on the patency rates of the AVFs after 72 h and over a period of six months. In any case, further researches on the effects of antiplatelet drugs on AVF patency is recomended.
